# Combining Longitudinal Data From Different Cohorts to Examine the Life-Course Trajectory

**DOI:** 10.1093/aje/kwab190

**Published:** 2021-07-02

**Authors:** Rachael A Hughes, Kate Tilling, Deborah A Lawlor

**Keywords:** Avon Longitudinal Study of Parents and Children, Barry Caerphilly Growth Study, Born in Bradford Study, Christ’s Hospital School Study, life course, mixed-effects models, Promotion of Breastfeeding Intervention Trial, repeated measures

## Abstract

Longitudinal data are necessary to reveal changes within an individual as he or she ages. However, rarely will a single cohort study capture data throughout a person’s entire life span. Here we describe in detail the steps needed to develop life-course trajectories from cohort studies that cover different and overlapping periods of life. Such independent studies are probably from heterogenous populations, which raises several challenges, including: 1) data harmonization (deriving new harmonized variables from differently measured variables by identifying common elements across all studies); 2) systematically missing data (variables not measured are missing for all participants in a cohort); and 3) model selection with differing age ranges and measurement schedules. We illustrate how to overcome these challenges using an example which examines the associations of parental education, sex, and race/ethnicity with children’s weight trajectories. Data were obtained from 5 prospective cohort studies (carried out in Belarus and 4 regions of the United Kingdom) spanning data collected from birth to early adulthood during differing calendar periods (1936–1964, 1972–1979, 1990–2012, 1996–2016, and 2007–2015). Key strengths of our approach include modeling of trajectories over wide age ranges, sharing of information across studies, and direct comparison of the same parts of the life course in different geographical regions and time periods. We also introduce a novel approach of imputing individual-level covariates of a multilevel model with a nonlinear growth trajectory and interactions.

## Abbreviations

ALSPAC: Avon Longitudinal Study of Parents and Children
BCG: Barry Caerphilly Growth
BiB: Born in Bradford
CHS: Christ’s Hospital School
CI: confidence interval
MI: multiple imputation
PROBIT: Promotion of Breastfeeding Intervention Trial

Life-course epidemiology aims to elucidate biological, behavioral, and psychosocial processes that operate across an individual’s life course to influence the development of disease risk (1). It requires repeatedly assessed data on risk factors and trajectories that reflect the underlying propensity of disease risk from infancy to adulthood (1). Trajectories of underlying disease propensity are necessary to establish ages of “peak” health for different diseases and the extent to which intervening during certain periods (e.g., periods of developmental origins or age-related decline) are likely to maximize population health. However, rarely will a single cohort study have data on a given risk factor or outcome throughout the life span.

An alternative to examining life-course trajectories in a single cohort is to model longitudinal data from multiple cohorts that cover different and overlapping periods of life. Examples include models of longitudinal blood pressure data from age 7 years to more than 80 years from 8 United Kingdom cohorts (2) and 4 United Kingdom studies (3), alcohol consumption from age 15 years to over 90 years from 9 cohort studies (4), and height and weight trajectories from birth to age 18 years using sparse longitudinal data from 16 studies (5). While these analyses demonstrated the potential of obtaining life-course trajectories by combining data from several cohorts that cover different periods of the life course, the authors did not describe the steps required to overcome the challenges involved in conducting this type of analysis.

In this paper, we investigate the challenges in combining data from independent cohorts with repeated measurements that cover different and overlapping periods of life. We describe in detail the steps needed to model life-course trajectories from several cohort studies. The key challenges are summarized in Web Table 1 (available online at https://doi.org/10.1093/aje/kwab190), and our Stata software code (StataCorp LLC, College Station, Texas) is presented in Web Appendix 1.

## ILLUSTRATIVE EXAMPLE

Our example examined the associations of sex, race/ethnicity, and parental education with children’s weight trajectories from birth to age 20 years. We analyzed data from 5 prospective cohort studies: the Avon Longitudinal Study of Parents and Children (ALSPAC) (6, 7), the Barry Caerphilly Growth (BCG) Study (8, 9), the Born in Bradford (BiB) Study (10), the Christ’s Hospital School (CHS) Study (11), and the Promotion of Breastfeeding Intervention Trial (PROBIT) (12, 13). The PROBIT cohort was from Belarus, and the remaining cohorts were from the United Kingdom. Collectively they covered different calendar periods from the 1930s to 2010. Children from multiple births were excluded because their growth patterns differ considerably from those of singletons. Parental education was a composite variable that took on all possible joint values of maternal highest educational attainment and paternal occupation (recorded around the time of birth, except for CHS). Data were harmonized across the cohorts.

We briefly describe each cohort study below; further details are provided in Web Appendix 2. Table 1 summarizes the cohort participants’ characteristics.

**Table 1:** Baseline Characteristics (%) of Children in 5 Cohort Studies After Data Harmonization

**Characteristic**	**Cohort**				
	**ALSPAC** ^**a**^ **, 1990–2012** **(*n* = 14,216)**	**BCG Study** ^**b**^ **, 1972–1979** **(*n* = 951)**	**BiB Study** ^**c**^ **, 2007–2015** **(*n* = 13,445)**	**CHS Study** ^**d**^ **, 1936–1964** **(*n* = 1,547)**	**PROBIT** ^**e**^ **, 1996–2016** **(*n* = 17,046)**
Age range, years^f^	0–20	0–5	0–6	9–18	0–16
Sex					
Male	51	54	52	100	52
Female	49	46	48	0	48
Missing data	0	0	0	0	0
Race/ethnicity					
White European	79	100^g^	39	100^g^	100^g^
South Asian	0	0	50	0	0
Other	4	0	8	0	0
Missing data	17	0	3	0	0
Maternal educational level^h^					
Left school at age 15 or 16 years	55	0	43	0	4
Left school at age 17 or 18 years	19	0	12	0	82
College degree or higher	11	0	21	0	14
Missing data	15	100	24	100	0
Paternal occupation^i^					
Class I or II	22	17	13	55	11
Class III	36	58	19	21	60
Class IV, V, or other	21	23	44	3	25
Missing data	21	2	24	21	4
No. of weight measurements^j^	157,000	12,737	78,110	89,070	205,864
No. of measurements per child^k^	10 (10)	14 (1)	5 (3)	57 (18)	13 (5)
Age at last measurement, years^k^	13.8 (13.1)	5 (0)	4.7 (2.7)	17.8 (1.5)	16.0 (1)

Abbreviations: ALSPAC, Avon Longitudinal Study of Parents and Children; BCG, Barry Caerphilly Growth; Bib, Born in Bradford; CHS, Christ’s Hospital School; PROBIT, Promotion of Breastfeeding Intervention Trial.

^a^ South West England.

^b^ South East Wales.

^c^ Center of North England.

^d^ South East England.

^e^ Republic of Belarus.

^f^ Values are expressed as range.

^g^ These cohorts had no record of race/ethnicity. On the basis of the populations from which they were recruited, we assigned all participants to White European race/ethnicity.

^h^ “Left school at age 15 or 16 years” in the United Kingdom means that the mother's highest academic qualification was either a Certificate of Secondary Education, Ordinary Level, or a General Certificate of Secondary School Education. “Left school at age 17 or 18 years” means the mother's highest academic achievement, generally, was the Advanced Level (A-Level). “College degree or higher” means the mother's highest academic achievement was either an undergraduate academic degree (awarded by colleges and universities upon completion of ≥3 years of study) or a postgraduate qualification (such as a master's-level or doctoral degree).

^i^ Social class I or II: professional or managerial occupation; social class III: intermediate occupation; social class IV or V or other: routine or unskilled occupation, or all other occupations (not categorized above).

^j^ Values are expressed as total number.

^k^ Values are expressed as median (interquartile range).

### ALSPAC cohort

All pregnant women residing in a defined area in the South West of England with an expected date of delivery between April 1, 1991, and December 31, 1992, were invited to take part in ALSPAC (6, 7). The children of these pregnancies have been followed up since birth. Weight was recorded at multiple time points between birth and late adolescence (e.g., at birth, at 6 weeks, at 10, 21, and 48 months, annually between ages 7 and 11 years, and at the target ages 12, 13, 15, and 17 years) (14). These measurements were obtained from several sources: medical records, research clinics, and parental reports.

### BCG Study cohort

The BCG Study is a follow-up of a dietary-intervention randomized controlled trial of pregnant women and their offspring (9) which recruited pregnant women residing in 2 towns in South Wales between 1972 and 1974 (8). Birth weights were abstracted from hospital records, and weight measurements were recorded by study nurses who visited participants at 10 days, 6 weeks, and 3, 6, 9, and 12 months, and thereafter at 6-month intervals, resulting in a total of 14 measurements by age 5 years (9).

### BiB Study cohort

All pregnant women booked for delivery at the Bradford Royal Infirmary (Bradford, United Kingdom) between March 2007 and November 2010 who attended the oral glucose tolerance test clinic (offered to all women at 26–28 weeks’ gestation) were invited to take part in the BiB Study (10). The children from these pregnancies have been followed up since birth. Weight measurements were recorded at multiple time points between birth and midchildhood (e.g., birth and, on average, at 2 and 6 weeks, 8 months, and 4 and 6 years) (14). These measurements were obtained from several sources: maternity records, child health records, primary-care records, the national child measurement program, and researchers’ assessments.

### CHS Study cohort

Regular height and weight measurements were recorded in boys aged 9–18 years who attended the (all-male) Christ’s Hospital School in West Sussex, United Kingdom, between 1936 and 1969 (11). The students were measured 3 times per school term (i.e., 9 measurements per year) by the school’s medical officer (11).

### PROBIT cohort

Thirty-one maternity hospitals and associated polyclinics (outpatient clinics for routine health care) in the Republic of Belarus participated in PROBIT, a randomized controlled trial of the effects of a breastfeeding promotion intervention. Mother-infant pairs were recruited during their postpartum hospital stay between June 1996 and December 1997 (12, 13). Birth weight was abstracted from hospital records, and weight was measured at scheduled study visits at ages 1, 2, 3, 6, 9, and 12 months and 6.5, 11.5, and 16 years (15). In addition, weight measurements were abstracted from primary-care records.

## METHODS

Below we describe the steps needed to model life-course trajectories from several cohorts: pooling the data (challenge 1), specifying an appropriate model for multilevel data on a nonlinear growth process (challenge 2), selecting the model for a large volume of data (challenge 3), and accounting for missing outcome and covariate data (challenges 4 and 5, respectively).

### Challenge 1: data harmonization

Data harmonization is required when pooling heterogeneous data from different studies (16). For comparable but differently measured variables, such as race/ethnicity, data harmonization derives new “harmonized” variables by identifying common elements across all studies.

#### Continuous measurements and sex.

The continuous measurements, age and weight, only required conversion to the same units. Researchers in all 4 birth cohorts recorded assigned sex at birth. The CHS cohort was from an all-boys’ school.

#### Child’s race/ethnicity.

For the BCG, CHS, and PROBIT cohort studies, which did not record race/ethnicity, we assumed that all children were of White European origin because there were very few people from minority racial/ethnic backgrounds in these populations during the cohort recruitment periods. We harmonized the BiB variable to the categories “South Asian” and “White European” (representing 50% and 39% of the cohort, respectively) and to “other” for the 8% of participants who were Black, mixed-race, and of other races/ethnicities (Web Table 2). Additionally, we harmonized the ALSPAC variable to the categories “White European” (79% of the cohort) and “other” (for the 4% of participants from Black African/Caribbean, South Asian, Chinese, mixed-race/ethnicity, and other racial/ethnic backgrounds (17)).

#### Maternal education.

Since qualifications can differ between countries and between calendar periods within a country (e.g., in 1987 the United Kingdom Ordinary-Level educational qualification was replaced by the General Certificate of Secondary Education), we defined the harmonized education variable by the number of years the mother had spent in school (Web Table 3). Researchers in the 2 older cohorts, BCG and CHS, did not measure maternal education, so all of those values were set to missing.

#### Paternal occupation.

The BCG and CHS studies classified paternal occupational social class on the basis of the United Kingdom Registrar General’s classification (18) (Web Table 4). For ALSPAC and BiB, paternal occupation was categorized using the United Kingdom Office for National Statistics’ Standard Occupational Classification (of 1990 and 2000, respectively). Using the National Statistics Socio-Economic Classification analytical classes (19) as a guide, we mapped the ALSPAC and BiB Standard Occupational Classifications to 3 occupational classes compatible with those of BCG and CHS (professional or managerial; intermediate; and routine or unskilled). The occupational categories of PROBIT cut across social classes (e.g., “service worker” included occupations ranging from professional to routine, and “manual worker” included skilled and unskilled manual occupations). Therefore, we used paternal occupation along with paternal highest educational attainment to classify PROBIT fathers’ jobs as “professional or managerial,” “intermediate,” or “routine or unskilled.”

### Challenge 2: accounting for the dependence structure of the data

The data had a nested 3-level structure (repeated measurements nested within an individual and individuals nested within a cohort). There are 2 main approaches for modeling multilevel data: marginal models (e.g., generalized estimating equations models) and mixed-effects models (also known as random-effects, multilevel, and hierarchical models) (20, 21). We focus on the linear mixed-effects model (hereafter referred to as a multilevel model), which can be used to make inferences about changes in the population mean response and to examine the data’s dependence structure (e.g., comparison of within-individual variability to between-individual variability).

Briefly, a multilevel model consists of “fixed effects” and “random effects” (22). The fixed effects describe the average relationship between the repeated measurements and time, which we shall call the “average growth trajectory.” The random effects are defined at each level of the data and describe the multiple sources of random variability in the data (e.g., random variability between cohorts, between individuals in the same cohort, and within an individual). Restricted maximum likelihood estimation is recommended because maximum likelihood estimation of the variance parameters (e.g., variance of a random effect) is biased downwards (22).

In our example, an important consideration was the small number of units at level 3 (i.e., 5 cohorts). Although there is no clear rule of thumb regarding sample size requirements, a simulation study investigating multilevel modeling of units with 1,000 observations each showed that a sample size of at least 25 level 2 units was required for reliable inference about the variance parameters (23). Therefore, we decided to fit a 2-level model (repeated measurements at level 1 and children at level 2) with cohort included as a categorical variable in the fixed effects with interactions between the cohort variable and the trajectory terms to allow each cohort to have its own average growth trajectory.

### Modeling the nonlinear growth trajectory

Most biological growth processes show nonlinear changes over time (14). Two common approaches to modeling nonlinear growth are fractional polynomials (24) and restricted cubic splines (25).

A restricted cubic spline divides the growth trajectory into distinct segments where adjacent segments are joined at knot points. A separate curve is fitted to each segment, where the first and last segments are restricted to be linear, and cubic polynomials are fitted for the interior segments.

A fractional polynomial models the entire trajectory using a single, special type of polynomial that can include logarithms (e.g., ${\mathrm{time}}^0=\ln (\mathrm{time})$), negative powers (e.g., ${\mathrm{time}}^{-2}=1/{\mathrm{time}}^2$), noninteger powers (e.g., ${\mathrm{time}}^{0.5}=\sqrt{\mathrm{time}}$), and repeated powers (e.g., ${\mathrm{time}}^2,{\mathrm{time}}^2$). The degree of the model indicates the number of fractional polynomial terms allowed (e.g., ${\mathrm{time}}^2$ is a 1-degree fractional polynomial, and ${\mathrm{time}}^2,{\mathrm{time}}^3$ is a 2-degree fractional polynomial).

In our example, we considered fractional polynomials of 1 and 2 degrees with powers of −2, −1, 0, 0.5, 1, 2, and 3 and restricted cubic splines with 3–7 knots. The knots were placed at percentiles (of age) to ensure there were adequate numbers of measurements between consecutive knots, as recommended by Harrell (25) and Durrleman and Simon (26). Our primary interest was the overall shape of the trajectory. However, when the location of the knots is of direct interest, subject-matter knowledge may be used to help position the knots (25, 26).

### Modeling the covariance structure

The covariance structure of the multilevel model accounts for the dependency within the data and is modeled via the random effects. Misspecification of the covariance structure can lead to incorrect standard errors for the fixed effects and invalid estimates of the random effects’ variance components (22).

At each level, the random effects are assumed to be normally distributed with means of 0 and an estimated variance-covariance matrix, where the diagonal elements are the variances of the random effects and the off-diagonal elements are pairwise covariances between the random effects. Many software implementations allow the analyst to place restrictions on the variance-covariance matrix (e.g., restrict a covariance to be 0). Typically, the random effects at each level are assumed to be mutually independent of the random effects at all other levels.

Including individual-level random effects for the intercept and slope term(s) allows the growth trajectories (i.e., starting positions and rate of change) to vary between individuals. Other variables, such as sex, can also be included as individual-level random effects.

The measurement-level random effects describe how an individual’s measurements vary about his/her growth trajectory. The simplest structure is a single random intercept which assumes the measurement-level variation is constant throughout. Alternatively, the model can allow the measurement-level variation to depend on age (known as complex level 1 variation (14)).

In our example, all models included individual-level random effects for the intercept and all trajectory terms. We did not put any constraints on the individual-level variance-covariance matrix (i.e., distinct variances and covariances were allowed).

Assuming a constant measurement-level variance was implausible for our data because of the wide age span (i.e., weights at age 18 years were more variable (e.g., 45–75 kg) than birth weights (e.g., 2.0–4.5 kg)). Therefore, we allowed for complex level 1 variation, comparing 2 approaches: 1) including an intercept and a linear term for age as measurement-level random effects and 2) dividing the age range into distinct segments and specifying a measurement-level random intercept for each segment, where measurement-level variance can vary across the segments but is assumed to be constant within each segment. For both approaches, we restricted all pairwise covariances (of the measurement-level variance-covariance matrix) to be 0.

### Challenge 3: model selection across multiple studies

Applying model selection separately within each cohort can bias selection toward simple functional forms, since single studies may lack the statistical power for selection of complex functional forms (27, 28). Conversely, conducting model selection on the combined data from all studies may be impractical because of the large volume of data.

We considered 2 alternative approaches: 1) performing model selection on the summed likelihood across the cohorts (i.e., the same model is fitted separately to each cohort and the likelihoods are summed across the cohort) (29) and 2) using a data-splitting approach (25, 30) in which model selection is conducted on a random sample of individuals from the combined data, where the proportion of individuals from each cohort is the same as per the combined data. An advantage of this second approach is that the remaining data (i.e., the unselected individuals) can be used as a “validation data set” to further evaluate model fit (25, 30).

Selection criteria used to compare nonnested models include Akaike’s Information Criterion, the Bayesian Information Criterion, and the mean squared prediction error (see Web Appendix 3 for further information).

The summed likelihood approach requires the same model to be fitted to each cohort separately. While we were able to fit the same fractional polynomial model to each cohort, it was not possible to fit the same restricted cubic spline model to cohorts with differing age ranges (e.g., to fit a model with knot points positioned at the same ages to cohorts with age ranges birth to 5 years and 9--11 years). Therefore, we used the random sample approach and selected the top 2 fractional polynomial and restricted cubic spline models based on a random sample of 15,000 children, and then compared the fit of these 4 models using the validation data set.

To reduce the number of candidate models, we conducted model selection in 2 stages. At stage 1 we selected the form of the nonlinear growth trajectory, where all models included the intercept and trajectory terms as fixed effects and individual-level random effects, and the measurement-level random effects were 2 independent random intercepts for the age periods ≤2 years and >2 years. At stage 2 we selected the measurement-level covariance structure of the best-fitting model from stage 1.

### Inclusion of covariates

In addition to the cohort variable, we included the covariates child’s sex, child’s race/ethnicity, and parental education as fixed effects, and for each covariate we included interactions between the covariate and the trajectory terms. These fixed effects were added to the best-fitting multilevel model. Note that we excluded interaction terms from the model that were not supported by the data (e.g., interactions between the South Asian category and the spline terms for ages ≥5 years).

### Challenge 4: accounting for missing outcome data

Likelihood estimation of multilevel models utilizes all observed repeated measurements and is not biased by the missing data when differences between the observed and missing data can be explained by associations with the observed outcome and covariate data (known as data that are missing at random (31)). In the absence of auxiliary information (i.e., data on variables not included in the multilevel model), this likelihood-based approach makes the same assumption about the missing data as standard implementations of multiple imputation (MI) and will yield standard errors with the same or greater precision (31, 32). See Web Appendix 4 for comments on measuring missing outcome data.

For ALSPAC, the age of last measurement indicated that a sizeable proportion of the cohort exited the study prematurely, which was also reflected in the relatively wide variability in the number of measurements per child (Table 1). In comparison, PROBIT, which had a similar age range to ALSPAC, had lower levels of dropout and lower variability in the frequency of measurement. The BCG Study was originally a trial with a prescribed measurement schedule, which reflects the high levels of participant retention and near-constant number of measures per child. CHS was a school-based study, so participant retention was high. Despite its prescribed measurement schedule, there was sizeable variation in the frequency of measurements (lower and upper quartiles were 49 and 67, respectively). This variability was mainly due to children entering the study (i.e., enrolling at the school) at different ages, with 47% of children enrolling before age 11 years, 52% enrolling between ages 11 and 12 years, and 1% enrolling at age 13 years or older. Since our example did not have any auxiliary information, we decided not to impute the outcome data.

### Challenge 5: accounting for missing covariate data

Combining data across cohorts raises the problem of systematically missing covariate data (i.e., covariate information is missing for all participants in a cohort because it was not measured (33)). Restricting the analysis to participants with observed data on all covariates, which we shall call “complete covariate analysis,” and using MI are 2 approaches with which to account for missing data. The choice of approach will depend on the missing-data setting (32). Specialized MI methods (e.g., see references 34–38) and software (e.g., jomo (39) and Stat-JR (40)) are required when the main analysis uses a multilevel model (32, 41).

In our example, there were missing data on the individual-level covariates race/ethnicity and parental education (both components: maternal education and paternal occupation) (Table 1). The discussion below excludes the missing data on child’s race/ethnicity in BCG, CHS, and PROBIT, as we assumed all children in these cohorts were of White European origin.

We decided to use MI instead of complete covariate analysis for 2 reasons. First, our investigations indicated that the chance of having complete covariate data depended on the observed outcome data; thus, a complete covariate analysis could have been biased by omitting children with missing data (32). Second, a complete covariate analysis would have been extremely wasteful, discarding all data on 17.4% of children: all 2,498 children in the CHS and BCG cohorts and 5,734 children in the ALSPAC and BiB cohorts. Factors identified as predictors of missingness and the missing values were included in the imputation model. Since the aim of our example was to illustrate our methodology, we only considered a small number of factors.

Neither jomo nor Stat-JR was suitable for our example because neither package allowed the measurement-level variance to vary with age, and an imputation model that assumed constant measurement-level variance failed to converge. Instead we adapted an imputation procedure developed for imputing level 2 data using summary measures of any level 1 data (36). Our adaption was an iterative procedure which incorporated summaries of the children’s growth trajectories in the imputation model. It accounted for the interactions of the main analysis because we used the same model to derive these child-specific growth trajectory summaries (see Web Appendix 4 for further details). We generated 25 imputed data sets and combined the multiple sets of results into a single inference using Rubin’s rules (42).

## RESULTS

The 5 cohorts contributed data from birth to age 20 years. The combined sample size was 47,205 children with 542,781 weight measurements (Table 1). The cohorts covered overlapping but differing age ranges with repeat weight assessments: birth to age 20 years for ALSPAC, birth to age 5 years for BCG, birth to age 7 years for BiB, ages 9–18 years for CHS, and birth to age 18 years for PROBIT. The CHS Study had the highest median number of weight measurements per child (at least quadruple that of the other cohorts), and the BCG Study had the second highest, with a median of 14 measures.

**Figure 1:**
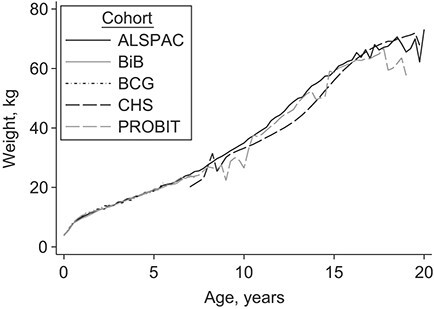
Observed mean weight trajectories of children in 5 cohort studies. ALSPAC, Avon Longitudinal Study of Parents and Children (1990–2012); BCG, Barry Caerphilly Growth Study (1972–1979); BiB, Born in Bradford Study (2007–2015); CHS, Christ’s Hospital School Study (1936–1964); PROBIT, Promotion of Breastfeeding Intervention Trial (1996–2016).

Figure 1 shows observed mean growth trajectories among the cohorts. During the first 6 months, the observed mean trajectory was approximately linear; otherwise the general trajectory shape was nonlinear: curving over in the first year, curving slightly under between ages 5 and 12 years, and starting to plateau around age 17 years. Compared with ALSPAC and PROBIT, the CHS cohort had a lower growth trajectory.

**Figure 2:**
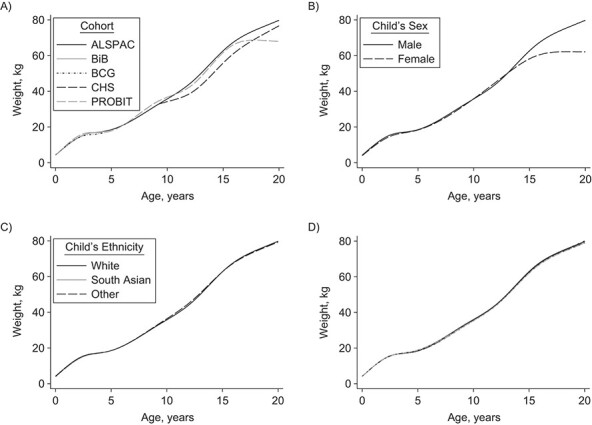
Predicted mean weight trajectories of children in 5 cohort studies according to cohort (A), child’s sex (B), child’s race/ethnicity (C), and parental education and employment status (D). Key for panel D—black long-dashed line: mother left school at age 15 or 16 years (i.e., the mother's highest academic qualification was either a Certificate of Secondary Education, Ordinary Level, or a General Certificate of Secondary School Education) and father had a routine/unskilled occupation; light gray short-dashed and dotted line: mother left school at age 17 or 18 years (i.e., the mother's highest academic achievement was Advanced Level (A-Level)) and father had a routine/unskilled occupation; dark gray solid line: mother had a college degree or higher and father had a routine/unskilled occupation; black short-dashed and dotted line: mother left school at age 15 or 16 years and father had an intermediate occupation; light gray solid line: mother left school at age 17 or 18 years and father had an intermediate occupation; dark gray long-dashed line: mother had a college degree or higher and father had an intermediate occupation; black solid line: mother left school at age 15 or 16 years and father had a professional/managerial occupation; light gray long-dashed line: mother left school at age 17 or 18 years and father had a professional/managerial occupation; dark gray short-dashed and dotted line: mother had a college degree or higher and father had a professional/managerial occupation. The solid black line in each graph denotes the predicted mean weight trajectory for the reference participants: White boys from the ALSPAC cohort with a mother who left school at age 15 or 16 years and a father whose occupation was classified as professional or managerial. ALSPAC, Avon Longitudinal Study of Parents and Children (1990–2012); BCG, Barry Caerphilly Growth Study (1972–1979); BiB, Born in Bradford Study (2007–2015); CHS, Christ’s Hospital School Study (1936–1964); PROBIT, Promotion of Breastfeeding Intervention Trial (1996–2016).

The results of the model selection procedure and a summary table of the components of the final model are provided in Web Appendix 5 and Web Table 5, respectively. The appropriateness of a linear slope in the early months of life is supported by the observed mean trajectory (Figure 1) and by the low values for the mean squared prediction error when fitting the restricted cubic spline with 7 knots (see Web Table 6) and has been reported elsewhere (14). Figure 2 shows predicted mean weight trajectories, using the MI point estimates of the final model, between children of different cohorts, sexes, racial/ethnic groups, and parental education groups (in each case holding all other covariates constant). Weight trajectories were very similar between the cohorts in the first 4 years of life. Between ages 10 and 15 years, children from ALSPAC were heaviest, those from PROBIT in between, and those from CHS lightest (e.g., at age 15 years, the predicted mean difference in weight between children from PROBIT and ALSPAC was −0.93 kg (95% confidence interval (CI): − 0.49, −1.37) and that between children from CHS and ALSPAC was −6.88 kg (95% CI: − 6.23, −7.52)). By age 20 years, the difference in weight between children from CHS and ALSPAC had narrowed (predicted mean difference = − 3.05 kg, 95% CI: − 1.80, −4.31). The marked plateauing effect after age 15 years for PROBIT could have been due to its limited number of measurements between ages 15 and 20 years. The weight trajectories of boys and girls were similar until adolescence and started to diverge after age 15 years. There were very little differences between children of different race/ethnic backgrounds or between those whose parents had different educational levels.

## DISCUSSION

Ideally, life-course epidemiology should use data from repeated assessments carried out from the time when participants were in utero or born to old age. The oldest birth cohorts currently available are from the 1950s/1960s (43, 44), meaning even they only go up to midlife. Those 2 cohorts also lack very detailed repeat measurements even of weight and height, with substantial gaps between childhood and later adult measures. An alternative is to combine cohorts that cover different but slightly overlapping life periods to understand the impact of developmental/early-life exposures on outcome trajectories across the life course (2). We have illustrated the key challenges in the use of this alternative approach and the fact that it allows not only examination of a longer life span but also direct comparison of the same parts of the life course in different geographical regions and time periods. We have also introduced a novel approach of imputing individual-level covariates of a multilevel model with a nonlinear growth trajectory and interactions.

In order to cover a life course, it is usually necessary to combine data from heterogeneous cohorts, in contrast to a meta-analysis of longitudinal studies (e.g., that of O’Neill et al. (45)), which selects cohorts for analysis based on their similarity of design. This raises the question of how to interpret results in terms of the target population. Because our aim is to explore meaningful trajectories across the life span, we are by definition assuming that all of the studies reflect the same underlying population. Thus, we might consider the results from our illustrative example to relate to a target population of all people, with the trajectories reflecting change from birth to age 20 years, despite differences in geography and race/ethnic distributions between studies. However, heterogeneity between cohort studies will assist in determining whether an overall target population is a reasonable concept here or not (46).

It is also necessary to make some modeling assumptions that are common to all cohorts in order to gain information over separate modeling of each cohort. This requires the analyst to make decisions on which common assumptions are plausible and which notable differences between the cohorts must be accommodated in the model. In our example, the weight trajectories of each cohort had the same shape, so it was plausible to model all of the data using the same nonlinear trajectory. At the same time, we had to allow for important differences, such as a lower mean trajectory for CHS than for ALSPAC and PROBIT. Because of differences between the cohorts, we did not use the model to predict mean trajectories beyond the data (e.g., for children aged ≥5 years from a South Asian background).

Data harmonization is required when pooling heterogeneous data from different studies. By necessity, the level of detail in the harmonized variable is often determined by the cohort with the most simplified definition of the variable. In addition, even after harmonization the interpretation of the harmonized variable may differ between cohorts (e.g., the meaning of maternal education may change over time or geography).

In meta-analyses, MI has been proposed as a solution for systematically missing covariate data (33, 47, 48), and in a study examining early- and midlife risk factors for cardiovascular disease in 4 pooled cohorts, al Hazzouri et al. (49) imputed values for exposure variables on which data were not available in younger cohorts. In our example, we multiply imputed the systematically missing data on maternal education but used a simpler approach for race/ethnicity, since population demographics at the time indicated that almost of all of the participants would have been of White European origin. For cohorts with greater racial/ethnic diversity, external population-level information could be utilized to multiply impute race/ethnicity as part of a sensitivity analysis (50).

Caution is needed when interpreting parameters common to all cohorts. In our example, the trajectories of boys and girls appeared to diverge from age 15 years onwards. While this may be a real phenomenon, we must also consider that this pattern may have been unduly influenced by the CHS cohort, which was the only cohort to cover the time period from the 1930s to the 1950s and which included data on boys only; its weight trajectories would probably have differed from those of boys from later periods because of changes in lifestyle and increases in average height.

In summary, careful analysis can harmonize and bring together information from different cohorts to inform studies of life-course trajectories. Our approach can allow investigators to leverage data from several cohorts to obtain more information on life-course trajectories and can help them examine heterogeneity between cohorts to shed light on influences on those trajectories.

## Supplementary Material

Web_Material_kwab190
